# *Phragmites australis* (Cav.) Trin. ex Steud. Extract Induces Apoptosis-like Programmed Cell Death in *Acanthamoeba castellanii* Trophozoites

**DOI:** 10.3390/plants11243459

**Published:** 2022-12-09

**Authors:** Hương-Giang Lê, Ji-Su Choi, Buyng-Su Hwang, Yong-Tae Jeong, Jung-Mi Kang, Tuấn-Cường Võ, Pyo-Yun Cho, Young-Kyung Lee, Won-Gi Yoo, Yeonchul Hong, Young-Taek Oh, Byoung-Kuk Na

**Affiliations:** 1 Department of Parasitology and Tropical Medicine, Institute of Health Sciences, Gyeongsang National University College of Medicine, Jinju 52727, Republic of Korea; 2 Department of Convergence Medical Science, Gyeongsang National University, Jinju 52727, Republic of Korea; 3 Nakdonggang National Institute of Biological Resources, Sangju 37242, Republic of Korea; 4 Department of Parasitology and Tropical Medicine, School of Medicine, Kyungpook National University, Daegu 41944, Republic of Korea

**Keywords:** *Phragmites australis* (Cav.) Trin. ex Steud. extract, amoebicidal activity, *Acanthamoeba castellanii*, programmed cell death, apoptosis, autophagy

## Abstract

Acanthamoeba keratitis (AK) is an infectious ocular disease which is difficult to diagnose correctly and cure. Development of an effective and safe therapeutic drug for AK is needed. Our preliminary screening of more than 200 extracts from wild plants collected in Korea suggested the potential amoebicidal activity of *Phragmites australis* (Cav.) Trin. ex Steud. extract (PAE) against *Acanthamoeba* species. Here, we aimed to analyze the amoebicidal activity of PAE on *Acanthamoeba* and its underlying amoebicidal mechanism. PAE induced amoebicidal activity against both *A. castellanii* and *A. polyphaga* trophozoites, while it showed low cytotoxicity in human corneal epithelial cells (HCE-2) and human retinal pigment epithelial cells (ARPE-19). Transmission electron microscopy analysis showed subcellular morphological changes, such as increased granules, abnormal mitochondria, and atypical cyst wall formation, in the PAE-treated *A. castellanii*. Fluorometric apoptosis assay and TUNEL assay revealed apoptosis-like programmed cell death (PCD) in the PAE-treated *A. castellanii*. The PAE treatment increased reactive oxygen species production and reduced mitochondrial membrane potential in the amoeba. The enhanced expression of autophagy-associated genes was also detected. These results suggested that PAE exerted a promising amoebicidal effect on *A. castellanii* trophozoites via the PCD pathway. PAE could be a potential candidate for developing a therapeutic drug for AK.

## 1. Introduction

*Acanthamoeba* species are free-living amoebae widely distributed in diverse natural environments. Several species of amoebae can infect mammals, including humans, and cause diseases, such as acanthamoeba keratitis (AK) and granulomatous amebic encephalitis (GAE). They also act as reservoirs for pathogenic bacteria, including *Legionella* species and *Mycobacterium* species [1,2]. AK is the most frequent *Acanthamoeba* infection in humans and can lead to vision loss due to severe corneal inflammation and ulceration [3,4]. Individuals who have corneal trauma and contact lens wearers with unsanitary habits are at a high risk for AK. Global AK cases have continuously grown due to the increased number of contact lens wearers [3,4,5,6]. When the amoeba infects the corneal epithelium, it invades the corneal stroma and causes inflammation and severe damage, which is often associated with prolonged treatment course and poor visual outcomes even after treatment [7]. The acute and early diagnosis of AK is essential for proper treatment, but it is often complicated due to nonspecific clinical signs and symptoms, resulting in the misdiagnosis of AK as other viral, bacterial, or fungal infections [8]. Even when a correct and early diagnosis is made, the treatment of AK is often difficult and not always effective. Chlorhexidine and 0.02% polyhexamethylene biguanide (PHMB) are the most common drugs applied to treat AK, but clinical isolates of *Acanthamoeba* with resistance to PHMB or other drugs have been reported [9,10], and PHMB can be toxic to human corneal cells [11,12]. Alternative combinations of antibacterial, antifungal, and aromatic diamidines have also been used to treat AK, but their treatment efficacy is not promised [13]. Therefore, the development of safe and effective therapeutic agents to cure AK is urgently needed.

Plants and natural products originating from medicinal plants have been used for managing or treating diverse diseases for many years in human history. Their promising roles in overcoming the current need and medicinal chemistry of natural products represent an attractive approach for the discovery and development of new therapeutic agents. They also have been widely used in many countries as traditional medicine in various types or formulas, such as crude extracts, infusions, or plasters, as they have the merits of being affordable and easy to use. Natural products also serve as important sources of raw materials or bioactive compounds for therapeutic purposes [14], but the ongoing scientific evaluation of their properties, efficacy, and safety as therapeutic drugs is needed. Recently, approaches to finding natural compounds in diverse natural resources with amoebicidal activity against *Acanthamoeba* have been performed [15,16,17,18,19,20,21,22,23,24,25]. These studies demonstrated the substantial amoebicidal or anti-amoebic effects of natural compounds or plant extracts against pathogenic *Acanthamoeba* species and suggested their potential application as therapeutic candidates for AK.

*Phragmites australis* (Cav.) Trin. ex Steud., a common reed, is one of the globally distributed and often dominant species in freshwater wetlands [26]. Although this plant has been traditionally used as food-making helper and spice in certain areas, the pharmacological effect and medical usefulness of the plant is poorly understood. Recently, potential anti-cancer, anti-bacterial, and anti-viral effects of *P. australis* (Cav.) Trin. ex Steud. have been reported [27,28,29]. In our pilot study involving the mass screening of more than 200 extracts from wild plants collected in Korea, we found that an extract of *P. australis* (Cav.) Trin. ex Steud. showed a potential amoebicidal activity for the pathogenic *Acanthamoeba* species. In this study, we analyzed the amoebicidal effect of the reed shoot of *P. australis* (Cav.) Trin. ex Steud. extract (PAE) against *Acanthamoeba* trophozoites and investigated the underlying molecular mechanism of the amoebicidal activity of the extract. PAE showed a promising amoebicidal activity against *Acanthamoeba* spp. and very low cytopathic effects in human cell lines. PAE induced subcellular morphological changes in the amoeba trophozoites, which could lead to the programmed cell death (PCD) of *Acanthamoeba*. These findings suggest that PAE could be a potential candidate for the development of a novel therapeutic drug for AK.

## 2. Results

### 2.1. PAE Showed Amoebicidal Activity against A. castellanii and A. polyphaga

**Figure 1:**
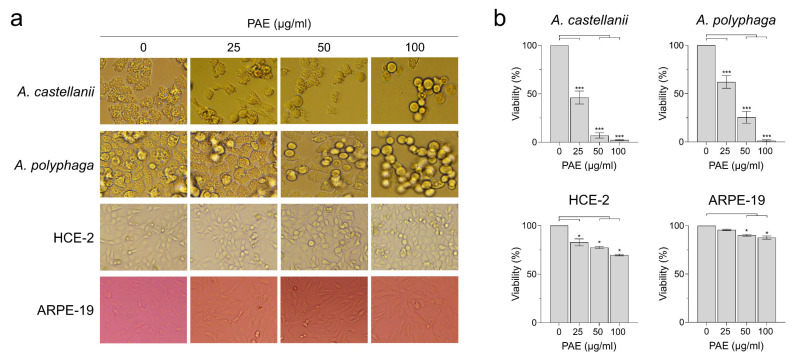
Amoebicidal activity of PAE against *Acanthamoeba* trophozoites. Different concentrations of PAE (0, 25, 50, or 100 μg/mL of final concentration) were added to *A. castellanii* and *A. polyphaga* trophozoites and incubated for 48 h. The amoebicidal activity of PAE was measured using microscopic examination (**a**) and the CellTiter-Blue^®^ Cell viability assay (**b**). HCE-2 and ARPE-19 cells were also included in the assay to evaluate the potential cytotoxicity of PAE in human cell lines. Both *Acanthamoeba* species show morphological changes upon treatment with PAE in a dose-dependent manner. PAE does not cause significant morphological changes in HCE-2 and ARPE-19 cells. The viability of the amoebae and human cells is presented as a percentage relative to the negative controls not treated with PAE. The results are shown as the mean and standard deviation (error bar) of each assay obtained using three independent assays. *** *p* < 0.0001, * *p* < 0.01.

To evaluate the amoebicidal activity of PAE, *A. castellanii* and *A. polyphaga* trophozoites were treated with different concentrations of PAE, and the morphological changes and viability of the amoebae were analyzed. PAE showed significant amoebicidal activity against *A. castellanii* and *A. polyphaga* in a dose-dependent manner (Figure 1). PAE induced morphological changes, such as rounded shapes and size reductions, resulting in a significant decrease in viability. However, PAE did not induce morphological changes in HCE-2 and ARPE-19 cells and did not affect the viability of the cells greatly, although a partial cytotoxic effect was detected in both cell lines at high concentrations of PAE (Figure 1). These results suggested that PAE showed selective amoebicidal activity against *A. castellanii* and *A. polyphaga* trophozoites, with low cytopathic effects in human HCE-2 and ARPE-19 cell lines.

### 2.2. PAE Induced Changes in the Ultrastructural Morphology of A. castellanii

**Figure 2:**
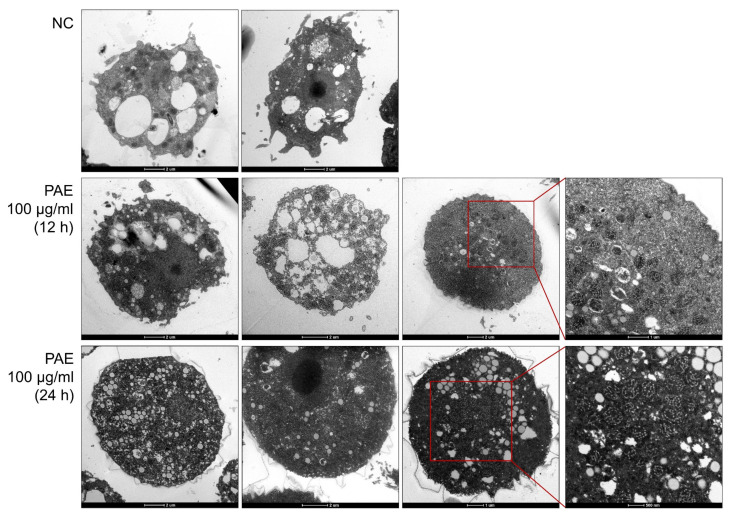
Transmission electron microscopy. *A. castellanii* trophozoites treated with PAE show ultrastructural morphological changes, such as the degradation of cellular organelles and the appearance of dense granules or blebs. Partial cytoplasmic shrinkage and mitochondrial deformation with increased numbers of abnormal shapes are also observed in the PAE-treated amoebae. Atypical cyst wall formation, with the absence of typical double-layered cyst walls, is also detected in the amoebae after 24 h of treatment with PAE. Autophagy-like structures are also found in the PAE-treated amoebae. NC, negative control amoebae treated with the same concentration of DMSO without PAE. Magnification 6000× or 10,000×.

Ultrastructural morphological changes in *A. castellanii* were observed upon treatment with PAE. PAE induced morphological changes in the amoebae at different time points (Figure 2). Partial degradation of cellular organelles and the appearance of intracellular compartments, such as dense granules or blebs, were detected in the PAE-treated amoebae. Mitochondrial deformation with increased numbers of abnormal mitochondria was also observed in the PAE-treated amoebae. Some amoeba cells also showed partial cytoplasmic shrinkage and destruction of the membranes. PAE induced the formation of cyst walls in the amoebae after 24 h of treatment, but they were atypical or immature, with the absence of typical double-layered cyst walls. Autophagic vacuoles, which are autophagy-like structures, were also found in the PAE-treated amoebae.

### 2.3. PAE Induced Apoptotic Death in A. castellanii

**Figure 3:**
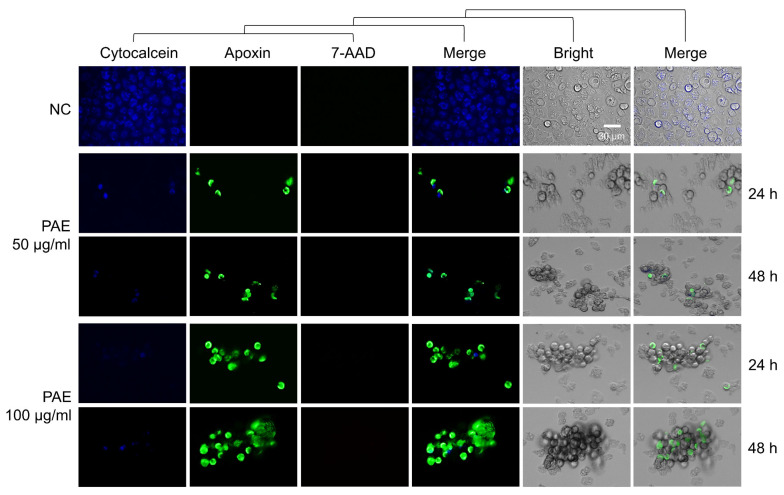
Apoptosis/necrosis assay. Dual-fluorescence staining assay was performed on *A. castellanii* trophozoites treated with 50 or 100 μg/mL of PAE for 24 or 48 h. Apoptosis/necrosis assay was performed using the Apoptosis/Necrosis Detection Kit (Abcam, Cambridge, UK) according to the manufacturer’s instructions. NC, negative controls treated with the same concentration of DMSO without PAE for 48 h. The images were obtained using an EVOS M5000 Imaging System (Thermo Fisher Scientific, Waltham, MA, USA). CytoCalcein stains living cells with blue fluorescence. Green apoxin and 7-ADD stain apoptotic cells green and necrotic cells red. The images (magnification of 200×) are representative of the cell population observed in three individual experiments.

**Figure 4:**
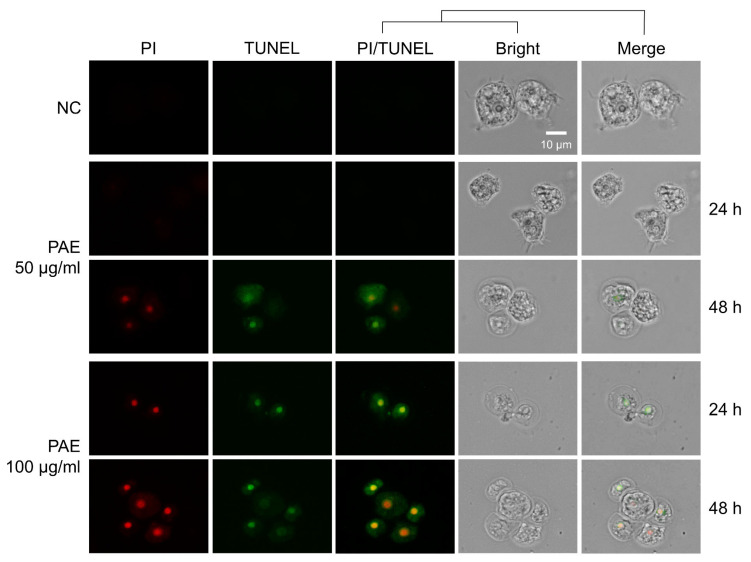
TUNEL assay. *A. castellanii* trophozoites were treated with 50 or 100 μg/mL of PAE for 24 or 48 h. The TUNEL assay was performed to detect DNA fragmentation in the amoebae upon treatment with PAE. The cells were counterstained with propidium iodide (PI, red) and TUNEL (green). NC, negative controls treated with the same concentration of DMSO without PAE. The images were obtained using an EVOS M5000 Imaging System at a magnification of 400×. The images are representative of the cell population observed in three individual experiments.

The induction of apoptosis in *A. castellanii* upon treatment with PAE was detected using a dual-fluorescence staining method. The PAE-treated *A. castellanii* showed a significant reduction in blue fluorescence (CytoCalcein for live cells) (Figure 3), whereas the control amoebae without PAE treatment showed blue fluorescence, indicating no apoptotic cells. Green fluorescence (apoxin for apoptotic cells) increased in the PAE-treated amoeba cells in time-dependent and dose-dependent manners. Red fluorescence (7-AAD), implying necrotic cells, was not detected in the PAE-treated *A. castellanii*. To further analyze the apoptotic events in the PAE-treated *A. castellanii*, DNA fragmentation was analyzed in the PAE-treated *A. castellanii* using the TUNEL assay. An increase in green fluorescence, indicating DNA fragmentation, was detected in the PAE-treated amoebae (Figure 4). These results collectively suggested that PAE induced the apoptotic death of *A. castellanii* trophozoites.

### 2.4. PAE Induced the Disruption of Mitochondrial Function in A. castellanii

**Figure 5:**
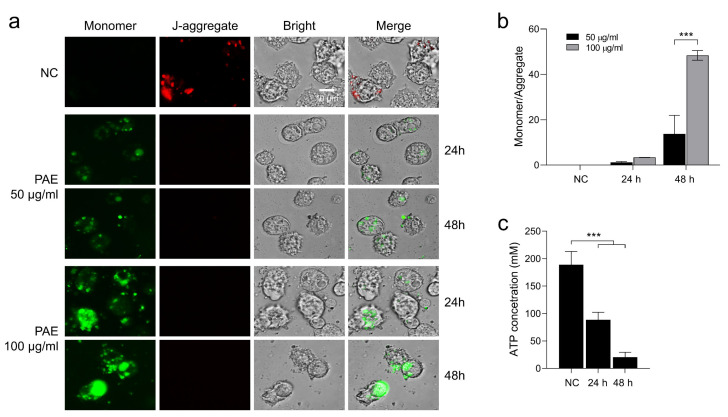
PAE induced the disruption of mitochondrial function in *A. castellanii*. (**a**) Mitochondrial membrane potential changes. *A. castellanii* trophozoites without PAE treatment (negative controls; NC) show only red fluorescence as JC-1 accumulates and aggregates in healthy mitochondria. PAE-treated amoeba cells display green fluorescence (monomer) caused by the collapse in the mitochondrial membrane potential. The images are observed using an EVOS M5000 Imaging System at a magnification of 400×. The images are representative of the cell population observed in three individual experiments. (**b**) Ratio of monomers and aggregates in amoebae treated with PAE. Time-dependent and dose-dependent changes in the ratio are observed. The mean and standard deviation (error bar) of the ratio are obtained using three independent assays. *** *p* < 0.0001. (**c**) ATP assay. ATP levels in the PAE-nontreated *A. castellanii* were compared to those in amoeba cells treated with PAE. A significant decrease in ATP production is observed in the amoebae treated with PAE (50 μg/mL) for 24 and 48 h. The mean and standard deviation (error bar) of each value are obtained using three independent assays. *** *p* < 0.0001.

PAE caused the collapse of the mitochondrial membrane potential in *A. castellanii*. Control amoebae without PAE treatment showed J-aggregate (JC-1) signals with red fluorescence, indicating high mitochondrial membrane potential (Figure 5a,b). When *A. castellanii* was treated with PAE, green fluorescence signals corresponding to JC-1 monomeric forms, implying the depolymerization of mitochondrial membrane potential through the inhibition of JC-1 agglomeration, increased in the cytoplasm of the amoebae (Figure 5a,b). JC-1 monomer signals increased with the PAE treatment in time-dependent and dose-dependent manners. Mitochondrial damage was also confirmed by measuring ATP generation in the amoebae. A consistent decrease in ATP levels was observed in the PAE-treated amoebae in a time-dependent manner (Figure 5c). These findings suggested that PAE induced the disruption of normal mitochondrial function in the amoebae.

### 2.5. PAE Induced Increased Intracellular ROS Production in A. castellanii

**Figure 6:**
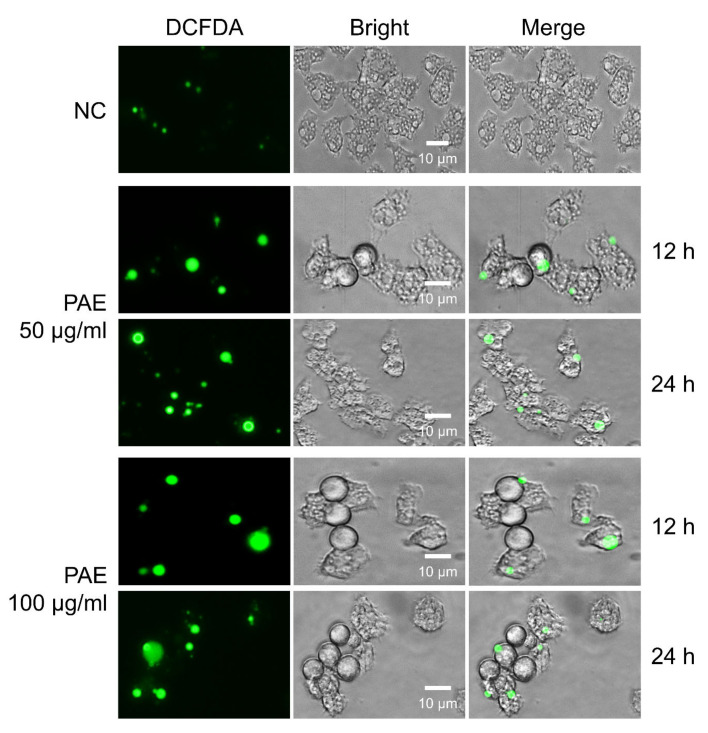
Increase in intracellular ROS in PAE-treated *A. castellanii*. Increased levels of intracellular ROS are detected in *A. castellanii* trophozoites treated with PAE. Amoebae were treated with different concentrations (50 or 100 μg/mL) of PAE for 12 or 24 h. The amoebae were incubated with DCFDA and observed with a fluorescence microscope. The images are representative of the cell population observed in three individual experiments. The images are observed using an EVOS M5000 Imaging System at a magnification of 400×. NC, negative controls without treatment of PAE.

Intracellular ROS generation in *A. castellanii* increased with the PAE treatment. Compared to the negative controls, the amoeba cells treated with PAE exhibited green fluorescence corresponding to ROS in time-dependent and dose-dependent manners (Figure 6).

### 2.6. PAE Induced Increased Expressions of Autophagy Markers

**Figure 7:**
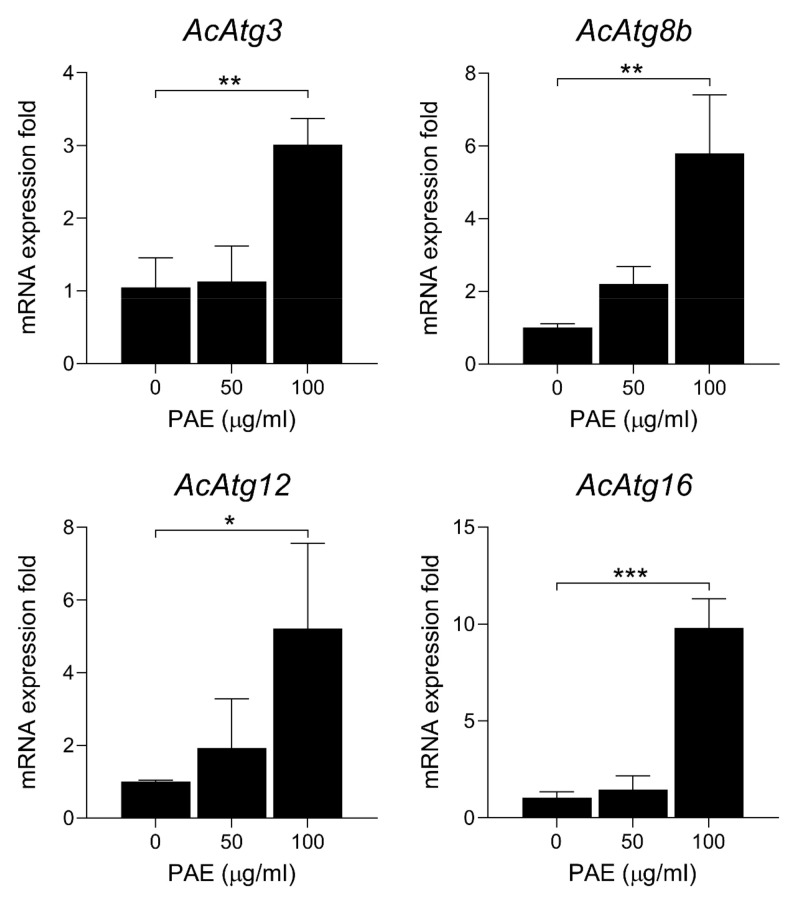
Expression profiles of autophagy-related genes. *A. castellanii* trophozoites were incubated with PAE (50 or 100 µg/mL) for 24 h. The amoeba cells were harvested, and total RNA was extracted. The expression of each gene was analyzed using qPCR. The expression fold of each gene at each time point was normalized to *A. castellanii* 18S rRNA, which was used as the internal control gene. The results are represented as the mean and standard deviation (error bar) of each assay obtained using three independent assays. *** *p* < 0.0001, ** *p* < 0.001, * *p* < 0.01.

To further analyze the association between the autophagic process and the PAE-induced amoebicidal effect, the expression patterns of autophagy-related genes were investigated in the *A. castellanii* treated with PAE. The expression of *AcAtg3*, *AcAtg8b*, *AcAtg12*, and *AcAtg16* increased significantly in the PAE-treated *A. castellanii* (Figure 7). The enhanced expression of these genes was detected in the *A. castellanii* treated with 50 µg/mL of PAE but was more significant in the amoebae treated with 100 µg/mL of PAE.

## 3. Discussion

In this study, we found the promising amoebicidal activity of PAE and investigated its potent amoebicidal mechanism in *A. castellanii*. PAE showed amoebicidal effects against *A. castellanii* and *A. polyphaga* trophozoites and induced obvious morphological changes in the amoebae, with no critical cytotoxic effects in HCE-2 and ARPE-19 human cell lines. To further understand the underlying amoebicidal mechanism of PAE, we selected *A. castellanii* as more biological information is known about this amoeba. The TEM analysis showed that PAE caused morphological changes, such as the appearance of unusual cytosolic vacuoles (dense granules or blebs), increased numbers of mitochondria with unusual shapes, and the formation of atypical cyst walls in *A. castellanii* trophozoites. Similar morphological changes, such as increased numbers of mitochondria, were also observed in *A. castellanii* cells treated with an autophagy inhibitor and chloroquine [30]. Our results suggested that PAE induced biochemical and physiological alterations in the amoeba that could lead to amoebic cell death.

Apoptosis is the process of programmed cell death (PCD), which occurs in immune reactions or when cells are damaged by diseases or toxic agents. Several biochemical and morphological changes take place in the cells during apoptosis, such as cell shrinkage, nuclear fragmentation, chromatin condensation, DNA fragmentation, increases in caspase activity, and excessive ROS production [31]. Apoptosis or apoptosis-like PCD has been reported in several protozoa under stress conditions and drug treatments [32,33,34,35]. It has also been reported that statins and voriconazole induced PCD in *A. castellanii* [36]. Evidence of apoptosis-like PCD was observed in the *A. castellanii* treated with PAE. Strong apoptotic signals (apoxin stain), DNA fragmentation, and increased ROS production were detected in the PAE-treated *A. castellanii*, suggesting that the amoebicidal activity of PAE was associated with apoptosis-like PCD in the amoebae.

Mitochondrial membrane potential (ΔΨm) plays a key role in maintaining the electrochemical potential of hydrogen ions required to synthesize ATP. The relation of ΔΨm and ATP level can influence the physiological activity of cells [37]. When mitochondria are damaged, decreased mitochondrial membrane potential results in a decrease in intracellular ATP production, and, thus, could lead to PCD [38,39]. Reductions in mitochondrial membrane potential and the subsequent decreases in ATP production have been proposed as a process inducing PCD in *Acanthamoeba* treated with plant extracts or compounds [23,24,40,41]. Several molecules in olive leaf extract revealed amoebicidal activity against *A. castellanii* Neff by inhibiting mitochondrial function [24]. Staurosporine from *Streptomyces sanyensis* activated PCD in *Acanthamoeba* via the mitochondrial pathway [40]. *Camellia sinensis* (green tea) brews also showed anti-acanthamoebic activity against *A. castelanii* trophozoites, probably via apoptosis-like pathway [21]. PAE also induced the depolarization of the mitochondrial membrane potential and resulted in a significant reduction in ATP production in *A. castellanii*, suggesting that PAE provoked mitochondrial damage, which might be caused by increased intracellular ROS in *A. castellanii*, and subsequently induced PCD. It has been proposed that desirable therapeutic agents for AK would induce apoptotic processes, rather than necrotic cell death, to minimize the host inflammatory response [36]. Our results suggested that PAE exerted amoebicidal effects against *A. castellanii* through apoptosis-like PCD pathways. Further analysis of PAE-induced apoptotic mechanisms in *A. castellanii* may offer the potential to subvert the amoebae and develop novel therapeutic agents. Due to the limited information on chemical compounds in PAE, it is not clear what compounds in PAE contribute to PCD of *A. castellanii*. Further isolation and characterization of active amoebicidal compounds from PAE are warranted.

Our TEM analysis results suggested that PAE induced encystation of *A. castellanii* trophozoites. The PAE-treated *A. castellanii* formed cyst walls, indicating that the amoebae were also likely to be under strong pressure to form encystation for survival. However, the PAE-treated *A. castellanii* failed to form mature cysts; rather, they formed atypical or immature forms of cysts with an absence of normal interspaces and double-layered cyst walls. An increased number of mitochondria with unusual shapes in the PAE-treated amoebae and the appearance of autophagy-like vacuoles in the cytoplasm also suggested that autophagy might be associated with *Acanthamoeba* cell death. To investigate autophagy-related processes in the PAE-treated *A. castellanii*, we analyzed changes in the expression levels of autophagy-related genes [42,43,44,45,46], which are known to relate to the encystation of *Acanthamoeba*. Increased *AcAtg3*, *AcAtg8b*, *AcAg12*, and *AcAtg16* transcripts were detected in the PAE-treated *A. castellanii*, suggesting that an autophagic process occurred in the amoebae, probably to protect against PAE-induced cellular stress. However, the atypical cyst walls did not protect the amoebae and the amoebae eventually died.

## 4. Materials and Methods

### 4.1. Preparation of PAE

Fresh shoots of *Phragmites australis* (Cav.) Trin. ex Steud. were collected at Ansan Reed Marshy Park (126.8423° E, 37.2707° N), Ansan, Korea, in April 2020. The plants were classified based on the classification keys [47]. The whole dried reed shoots were mechanically grounded using an electrical blender. A voucher specimen (NNIBRVP7884) was deposited at the Library of the Nakdonggang National Institute of Biological Resources. An ethanolic extract of the plant was prepared by macerating 3 kg of the grounded plants, extracting twice in 70% aqueous ethanol (3 H_2_O: 7 ethanol, *v*/*v*, 50 L) for 2 days at room temperature (RT), and then filtering through filter paper (CHMLAB, Terrassa-Barcelona, Spain). The alcohol in the sample was removed using a rotary vacuum evaporator. The crude extract was concentrated by incubating at 37 °C, and fully dried extract powder was obtained.

### 4.2. Cultivation of Acanthamoeba and Human Cell Lines

*Acanthamoeba castellanii* (ATCC-30868) and *A. polyphaga* (ATCC-30461) were axenically cultured and maintained in a peptone-yeast-glucose (PYG) medium at 25 °C [48]. Human corneal epithelium cells (HCE-2; ATCC CRL-11135) were cultured in a keratinocyte-serum free medium (Gibco, Grand Island, NY, USA) supplemented with 0.05 mg/mL of bovine pituitary extract (Gibco, Grand Island, NY, USA), 5 ng/mL of epidermal growth factor (Gibco), 1× human corneal growth supplement (HCGS, Gibco, Grand Island, NY, USA), and 1% penicillin/streptomycin (P/S, Gibco, Grand Island, NY, USA). Human retinal pigment epithelial cells (ARPE-19; ATCC CRL-2302) were cultured in Dulbecco’s Modified Eagle’s Medium (DMEM; Welgene, Daegu, Korea) supplemented with 10% heat-inactivated fetal bovine serum (FBS; Gibco, Grand Island, NY, USA) and 1% P/S (Gibco, Grand Island, NY, USA). The cells were incubated at 37 °C in a humidified incubator under a 5% CO_2_ atmosphere.

### 4.3. Amoebicidal Assay

Dried PAE was dissolved in 100% dimethyl sulfoxide (DMSO; Sigma, St. Louis, MO, USA) and prepared at a stock concentration of 100 mg/mL. The screening of amoebicidal activity was performed on a 96-well microplate. *A. castellanii* and *A. polyphaga* trophozoites in the PYG medium were seeded into a 96-well microplate (SPL Life Sciences, Seoul, Republic of Korea; 5 × 10^4^ cells/well) and incubated at 25 °C overnight. Different concentrations (0 to 100 μg/mL) of PAE were added to the *A. castellanii* and *A. polyphaga* trophozoites and incubated at 25 °C for 48 h. The edge wells were filled with a sterile phosphate-buffered saline (PBS, pH 7.4) to protect against evaporation. Morphological changes in the amoebae were observed microscopically at the indicated incubation times. The viability of the amoebae was analyzed using the CellTiter-Blue^®^ Cell viability assay (Promega, Madison, WI, USA), according to the manufacturer’s instructions, and calculated using GraphPad Prism 9.1.0 software (GraphPad Software, San Diego, CA, USA). Amoebae treated with 0.1% DMSO, which had the same concentration as the diluted PAE used to treat the amoebae and confirmed to be non-toxic, were used as the controls, representing 100% cell viability.

### 4.4. Cytotoxicity Assay for HCE-2 and ARPE-19 Cells

The potential cytotoxicity of PAE in HCE-2 and ARPE-19 cells was analyzed using a cell viability assay. The cells were seeded in a 96-well microplate (SPL Life Sciences, Seoul, Korea, 2 × 10^4^ cells/well) and incubated overnight until 80% confluent. Serially diluted PAE (25, 50, 100, and 150 µg/mL) was added to the cells and incubated for 48 h. Morphological changes in the cells were observed using microscopic examination. Cell viability was analyzed using the CellTiter-Blue^®^ Cell viability assay (Promega, Madison, WI, USA) according to the manufacturer’s protocol. Cells treated with 0.1% DMSO, which was confirmed to not alter normal morphology using microscopic examination, were used as positive controls with 100% cell viability.

### 4.5. Transmission Electron Microscopy (TEM)

The *A. castellanii* trophozoites treated with PAE (100 µg/mL) for 12 h and 24 h were harvested, washed three times with PBS, and pelleted by centrifugation at 200× *g* for 5 min. The amoeba cells not treated with PAE were also prepared using the same protocol as the negative controls. The amoeba cells were fixed with 4% ice-cold glutaraldehyde and post-fixed with 1% osmium tetroxide in 0.1 M of phosphate buffer (pH 6.9) for 1 h. The cells were rinsed in distilled water, dehydrated with increasing concentrations of ethanol, and embedded in epoxy resin (Embed-812; Electron Microscopy Sciences, Hatfield, PA, USA). The blocks were cut into ultrathin sections (90 nm) and transferred onto 200-mesh copper grids (Agar Scientific, Stansted, UK). The sections were stained with a 4% uranyl acetate-lead citrate solution. Subcellular morphological structures of the amoebae were examined using a Talos L120C Cryo Bio 120 kV Transmission Electron Microscope (Thermo Fisher Scientific, Waltham, MA, USA) at the Gyeongsang National University Center for Research Facilities (Jinju, Republic of Korea).

### 4.6. Fluorometric Apoptosis/Necrosis Assay

Apoptosis/necrosis in the PAE-treated *A. castellanii* trophozoites was assessed using the Apoptosis/Necrosis Detection Kit (Abcam, Cambridge, UK) according to the manufacturer’s instructions. Briefly, the amoeba cells (5 × 10^4^ cells/well) were seeded in a 96-well black/clear bottom plate (Thermo Fisher Scientific, Waltham, MA, USA) and incubated with PAE (50 or 100 μg/mL) at 25 °C for 24 or 48 h. Green apoxin, CytoCalcein, and 7-aminoactinomycin D (7-AAD) components were added to the PAE-treated *A. castellanii* cells and incubated at RT in the dark for 1 h. The amoeba cells were washed with PBS three times and the fluorescence signal was observed using a fluorescence microscope (EVOS M5000 Imaging System, Thermo Fisher Scientific, Waltham, MA, USA) in the GFP channel (Ex/Em = 490/525 nm) for apoxin green, the Texas Red channel (Ex/Em = 550/650 nm) for 7-AAD, and the DAPI channel (Ex/Em = 405/450 nm) for CytoCalcein Violet 450. The amoebae treated with the PYG medium containing 0.1% DMSO were used as the negative controls.

### 4.7. TUNEL Assay

*A. castellanii* trophozoites (10^6^ cells/well) were seeded in a 6-well plate (Thermo Fisher Scientific, Waltham, MA, USA), treated with different concentrations of PAE (50 or 100 µg/mL), and incubated at 25 °C for 24 h and 48 h. The cells were harvested and fixed in 3.7% formaldehyde at RT for 20 min. After two washes with PBS, the cells were incubated with 70% cold ethanol for 30 min. The cells were double-stained by terminal deoxynucleotidyl transferase dUTP end-labeling (TUNEL) and propidium iodide (PI) staining using the TUNEL fluorescein isothiocyanate (FITC) assay kit (Abcam, Cambridge, UK) following the manufacturer’s instructions. The amoebae treated with the PYG medium containing 0.1% DMSO were used as the negative controls. The cells were observed using a fluorescence microscope (EVOS M5000 Imaging System, Thermo Fisher Scientific, Waltham, MA, USA) in the GFP channel (Ex/Em = 490/525 nm) for TUNEL and the Texas Red channel (Ex/Em = 550/650 nm) for PI.

### 4.8. Mitochondrial Membrane Potential Assay

Changes in the electrochemical gradient across the mitochondrial membrane were measured using the JC-1 Mitochondrial Membrane Potential Assay Kit (Abcam, Cambridge, UK) following the manufacturer’s protocols. JC-1 accumulates in healthy mitochondria, aggregates, and emits red fluorescence, whereas collapsed mitochondria emit green fluorescence due to decreases in mitochondrial membrane potential. *A. castellanii* trophozoites (5 × 10^4^ cells/well) were seeded in a 96-well black/clear bottom plate (Thermo Fisher Scientific, Waltham, MA, USA) and incubated with PAE (50 or 100 μg/mL) at 25 °C for 24 or 48 h. The cells were washed with PBS, and JC-1 (1 μM) was added to each well and incubated at RT for 30 min in the dark. Green and red fluorescence were observed using a fluorescence microscope (EVOS M5000 Imaging System, Thermo Fisher Scientific, Waltham, MA, USA) in the GFP channel (Ex/Em = 490/525 nm) for aggregated JC-1 and the Texas Red channel (Ex/Em = 550/650 nm) for the JC-1 monomer. The amoebae treated with the PYG medium containing 0.1% DMSO were used as the negative controls.

### 4.9. Measurement of ATP Levels

Changes in ATP levels in the PAE-treated *A. castellanii* were measured using an ATP assay kit (Abcam, Cambridge, UK) according to the manufacturer’s instructions. The amoebae were incubated with different concentrations of PAE (50 or 100 μg/mL) and incubated at 25 °C for 24 or 48 h. The cells were rinsed with PBS, resuspended in 100 μL of ATP assay buffer, and homogenized. The supernatant was collected and incubated with ATP reaction mixture at RT for 30 min. Absorbance was measured at 570 nm using a Multiskan FC spectrophotometer (Thermo Fisher Scientific, Waltham, MA, USA). The amoeba cells not treated with PAE were used as the controls. The assays were performed in three independent replications. The results are presented as the mean ± standard deviation (SD) of three individual assays. Statistical analyses were performed using GraphPad Prism 9.1.0 (GraphPad Software, San Diego, CA, USA) and statistical significance was analyzed using one-way of variance (ANOVA) with Dunnett’s post hoc test. The differences in the mean values were considered statistically significant at *p* < 0.001.

### 4.10. Intracellular Reactive Oxygen Species (ROS) Assay

To analyze the generation of intracellular ROS in the PAE-treated *A. castellanii*, the DCFDA/H2DCFDA-Cellular ROS assay kit (Abcam, Cambridge, UK) was used. In brief, the amoebae were plated into a black 96-well microplate (Thermo Fisher Scientific, Waltham, MA, USA; 5 × 10^4^/well) and incubated at 25 °C overnight. The amoebae were treated with different concentrations of PAE (50 µg/mL or 100 µg/mL) and incubated for 12 h and 24 h, respectively. The amoebae treated with the PYG medium containing the same concentration of DMSO were used as the negative controls. After incubation, the amoeba cells were washed with PBS and stained with 2′,7′-dichlorofluorescein diacetate (DCFDA) solution (20 µM) at 25 °C in the dark for 45 min. The cells were washed with PBS three times and the fluorescence signal was observed using a fluorescence microscope (EVOS M5000 Imaging System, Thermo Fisher Scientific, Waltham, MA, USA) in the GFP channel (Ex/Em = 490/525 nm).

### 4.11. Quantitative Polymerase Chain Reaction (qPCR) for Transcripts of Autophagy-Related Genes

*A. castellanii* trophozoites (10^5^ cells/well) were cultured in a 12-well plate in the absence or presence of PAE (50 or 100 µg/mL) for 24 h. The amoebicidal effect in the PAE-treated amoebae was confirmed using microscopic examination. The amoeba cells were harvested, and total RNA was extracted using RNAiso Plus (Takara, Otsu, Japan) following the manufacturer’s protocol. The RNA concentration in each sample was measured using a spectrophotometry (DeNovix DS-11; Wilmington, DE, USA), equalized, and used for cDNA synthesis. A total of 3 µg of mRNA was converted to cDNA using RNA to cDNA EcoDry Premix (Clontech, Mountain View, CA, USA) according to the manufacturer’s protocol. Realtime quantitative polymerase chain reaction (qPCR) was performed with specific primers for *AcAtg3* (5′-GGAGGAGTTCGATGAAGGTGA-3′ and 5′-ATGAACACTTGGTTCGGCGTC-3′), *AcAtg8* (5′-AAGAGCGATGCCCCCGACATC-3′ and 5′-CCATCCTCGTCCTTGTACTTGG-3′), *AcAtg12* (5′-GGTGGCGCCTCCGCGTTGAAG-3′ and 5′-GCAATAGTTGATCACCAGCTGG-3′), and *AcAtg16* (5′-AGCTTGACTTCCATCACGCTGA-3′ and 5′-ACCAGCTTGCCCGAAGCAGTC-3′) using the Quant Studio 1 Real-time PCR System (Thermo Fisher Scientific). *Ac 18S rRNA* (5′-TCCAATTTTCTGCCACCGAA-3′ and 5′-ATCATTACCCTAGTCCTCGCGC-3′) was amplified as an internal control. The PAE-untreated amoebae were used as the negative controls. The validation of PCR primers targeting each gene was performed using conventional PCR, followed by agarose gel electrophoresis (Figure S1).

### 4.12. Statistical Analysis

All the above experiments were conducted in three independent replications. Statistical analysis was performed using GraphPad Prism 9.1.0 (GraphPad Software, San Diego, CA, USA). Statistical significance was analyzed using a one-way analysis of variance (ANOVA) with Dunnett’s post hoc test. A difference in mean values was considered statistically significant at *p* < 0.01.

## 5. Conclusions

This is the first report on the amoebicidal effect of PAE. Our study suggested that PAE induced remarkable morphological and biological changes in *A. castellanii* trophozoites and showed promising amoebicidal activity against the amoeba by inducing PCD-like mechanisms and autophagy-related pathways in the amoeba. However, it showed low cytotoxicity in human cell lines, such as HCE-2 and ARPE-19. PAE has the potential to be used for medical purposes and utilized as a source of anti-*Acanthamoeba* agent development. These findings provide a basis for the use of PAE as an alternative therapy and suggest an effective chemotherapeutic agent with less toxicity. The amoebicidal activity of PAE against *Acanthamoeba* cysts also needs to be further investigated. Further isolation and characterization of active compounds from PAE with amoebicidal activity are needed. Studies using in vivo models to evaluate the therapeutic effects of PAE or PAE-derived compounds should also be performed.

## Data Availability

The data presented in this study are available in this article.

## Supplementary Materials

The following supporting information can be downloaded at https://www.mdpi.com/article/10.3390/plants11243459/s1, Figure S1: Validation of PCR primers targeting autophagy-related genes of *A. castellanii*. Each gene was amplified using conventional PCR and analyzed using agarose gel electrophoresis.
