# Supplementary figures and images for: Phragmites australis (Cav.) Trin. ex Steud. Extract Induces Apoptosis-like Programmed Cell Death in Acanthamoeba castellanii Trophozoites
Source: Plants (Basel). 2022 Dec 9;11(24):3459. doi: 10.3390/plants11243459 (PMC9783201; doi:10.3390/plants11243459)

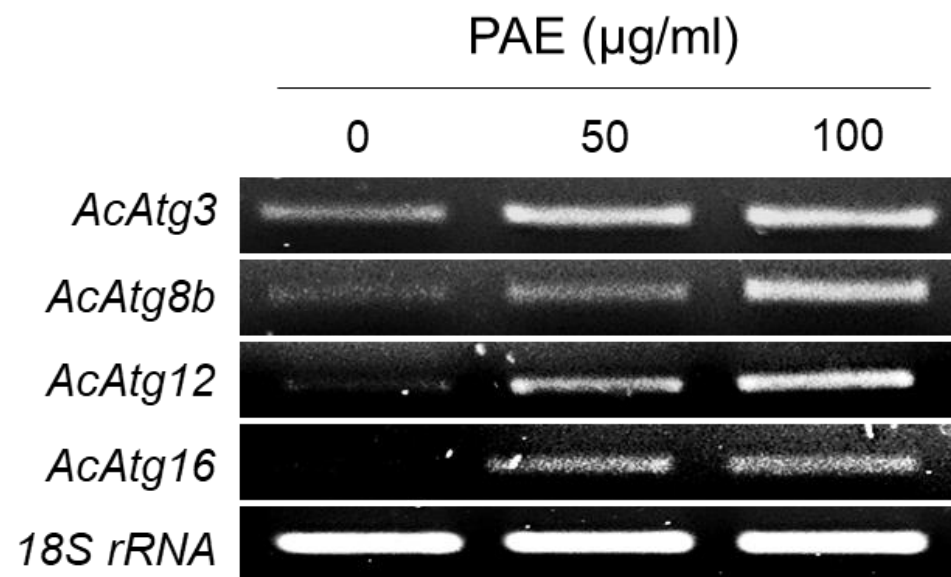

Supplement: Supplementary file 1 [file plants-11-03459-s001.zip › plants-2038122-supplementary.pdf]
